# Prevalence of Novel Psychoactive Substance (NPS) Use in Patients Admitted to Drug Detoxification Treatment

**DOI:** 10.3389/fpsyt.2020.00569

**Published:** 2020-07-07

**Authors:** Michael Specka, Thomas Kuhlmann, Jürgen Sawazki, Udo Bonnet, Renate Steinert, Monika Cybulska-Rycicki, Helmut Eich, Benita Zeiske, Antje Niedersteberg, Luzia Schaaf, Norbert Scherbaum

**Affiliations:** ^1^ LVR Hospital Essen Department of Addictive Behaviour and Addiction Medicine, Medical Faculty, University of Duisburg-Essen, Essen, Germany; ^2^ Psychosomatische Klinik Bergisch Gladbach, Bergisch Gladbach, Germany; ^3^ LVR Clinic Viersen, Viersen, Germany; ^4^ Castrop-Rauxel Evangelical Hospital, Castrop-Rauxel, Germany; ^5^ LWL-Klinik Münster, Münster, North Rhine-Westphalia, Germany; ^6^ LVR Clinic Langenfeld, Langenfeld, Germany; ^7^ Alexianer Hospital, Krefeld, Germany; ^8^ Alexius/Josef Hospital, Neuss, Germany; ^9^ LVR Clinic Düren, Düren, Germany

**Keywords:** novel psychoactive substances, pregabalin abuse, multiple substance use, drug dependence, drug detoxification, opiate dependents

## Abstract

**Background:**

About 15 years ago, a diverse group of new recreational psychotropic substances began to emerge, which were marketed for example as “legal highs,” “research chemicals,” or “designer drugs.” These substances were later subsumed under the label “Novel Psychoactive Substances” (NPS). Important NPS classes are cathinones, synthetic cannabimimetics, phenethylamines, and herbal drugs. The health care system for psychotropic substance use disorders (SUDs) traditionally focused on a few substances, such as alcohol, heroin, cocaine, amphetamines, or cannabis. Users of illicit substances often engage in polydrug use. However little is known about the prevalence of NPS use within the group of “classical” illicit substance users.

**Objective:**

We investigated lifetime and recent use of NPS and other drugs in patients who underwent in-patient detoxification treatment from illicit drugs in Germany.

**Methods:**

In a multicenter study with eight participating facilities, patients admitted to treatment underwent a standardized interview at admission, concerning their past and current substance use. The interview comprised classical substances of abuse, NPS, and rarely used substances such as LSD. In addition, participating sites had the opportunity to analyze their patients’ routine drug screenings by means of gas chromatography/mass spectrometry (GC/MS), which permitted detection of NPS.

**Results:**

Interviews from 295 patients could be analyzed. Most patients were opiate dependent and multiple substance users. About 32% reported use of synthetic cannabimimetics during lifetime, but usually only a few times. An important reason for their use was that NPS were not detected by drug testing in prisons or drug treatment facilities. Cathinones, herbal drugs or other NPS had rarely been used during lifetime. NPS use during the last 30 days before admission was nearly zero. This was confirmed by urine analysis results. In contrast, lifetime and current use of opiates, alcohol, cocaine, benzodiazepines, and cannabis was high. In addition, 18% reported of regular unprescribed pregabalin use during lifetime, and 20% had recently used pregabalin.

**Conclusion:**

Patients admitted to drug detoxification treatment showed multiple substance use, but this did not include NPS use. The diversion of legal medications such as pregabalin in this group is a serious concern.

## Introduction

The health care system for substance related disorders has traditionally been concerned with a limited number of psychoactive drugs, for example alcohol, opiates, cocaine, cannabis, and amphetamines, and to a much lesser degree with hallucinogenic drugs such as LSD, psilocybin, and others (1). About 15 years ago, the market for psychotropic substances saw the advent of a range of diverse substances, designed as legal alternatives to legally controlled drugs (2). These substances first gained prominence under labels such as “legal highs,” “herbal highs,” “research chemicals,” or “spice.” The term Novel Psychoactive Substances (NPS) which is used to describe these substances is not descriptive of their intrinsic properties. NPS is a label for substances imitating the effects of known psychotropic substances. Effects can resemble those of cannabis, of hallucinogens, or even of opioids.

The term NPS denotes, amongst other, synthetic cannabimimetics (agonists at the cannabinoid receptors), synthetic cathinones, which chemically resemble amphetamines or methamphetamine (for example mephedrone), phenetylamines as stimulants or hallucinogens. In addition, some authors also include ketamine and herbal products such as salvia divinorum. The early warning system of the European Union has documented the emergence of several hundred new NPS over the years (3).

NPS were initially not regulated by the law so that their possession and sale was legal. If a certain NPS substance was prohibited, it could be re-introduced after small chemical changes had been made. Legal regulations in response to this were introduced in several countries during the last few years. For example, the German “Neue psychoaktive-Stoffe-Gesetz” (NpSG) from November 2016 meant a far-reaching prohibition of complete groups of NPS and no longer of only singular substances. In the UK, the Psychoactive Substances Act (2016) was imposed, which criminalizes any NPS (4).

The most frequent NPS classes are associated with medical risks (1). The scientific literature has documented fatalities associated with or even attributable to the use of NPS. An analysis from 2015 of hospital emergency data by the European Drug Emergencies Network found that 9% of all drug‐related emergencies involved new psychoactive substances (5). Substances involved are often synthetic cannabinoids (e.g., “spice”) and synthetic cathinones (e.g., “bath salts”) (6). Synthetic cannabinoids can cause agitation, tachycardia, and arterial hypertension. There were emergency admissions with myocardial infarction, epileptic seizures, unconsciousness, aggressiveness, and psychiatric symptoms. Tryptamines, as 5-HT2A-receptor-agonists, have a hallucinogenic effect. Tryptamines have a low potential for being addictive, but there is a risk for the development of tolerance, and of cross tolerance with serotonergic substances. Phenetylamines can cause cardiovascular and psychiatric symptoms. There were documented emergency admissions with panic and agitation (1).

Prevalence of NPS use in the general population is seemingly low. In Germany, a general population survey on 18 to 64 year olds in 2015 estimated a 2.8% prevalence of lifetime NPS use, defined as “legal highs, research chemicals, bath salts, spice, or novel psychoactive substances, which could be available for example as herbal mixtures, powder, crystals, or pills.” The 12-month prevalence was 0.9%, and prevalence during the last 30 days was nearly zero (7, 8). Prevalence in the younger age groups of the general population is higher. Estimates from Europe suggest between 1% and 8% of school students have used NPS at some point (2). In an online survey, 6.7% of Dutch university students reported of NPS use (9). Even higher consumption rates were found in young attendees of electronic dance music events or nightclubs (10–12). Relevant rates of cathinone use have been found in several studies on adolescents and young adults (13).

It is yet unclear how much users of “classical” illicit psychotropic substances are attracted by NPS. In that population, multiple substance use is frequent and can include cocaine, opiates, cannabis, benzodiazepines, alcohol, and/or amphetamines. Use of NPS is not routinely assessed, for example in drug screenings. This could make NPS use attractive during detention, in residential homes or therapeutic facilities, while on parole, or in association with driving license issues.

Although administration of NPS is mainly *via* non-injecting routes (14), data from Hungary (15) and Scotland (16) suggest that persons who inject drugs might also inject NPS. In a sample of drug dependent and/or acutely intoxicated patients from hospitals in Paris and its suburbs (17), prevalence of NPS use was 29%, according to hair analysis. In that study, ketamine was subsumed under NPS and constituted more than half of the consumed substances. Users had used more than one NPS in about half of cases, mostly in combination with conventional drugs of abuse. In a study from Scotland, about 24% of patients with a substance use disorder (including alcohol) reported that they had consumed NPS before admission (4). By analysis of hair sampled from confirmed amphetamine users in Switzerland, a 37% rate of NPS users was found (18). In an interview study with clients from drug counseling centers in Germany, 6 out of 33 clients with opiate problems (18.2%), and 21 out of 48 polysubstance users (43.8%) reported of past NPS use, in the vast majority cannabimimetics like “spice” (19). In addition, NPS were detected in 13% of urine samples from opiate substituted patients in Sweden, but patients were rarely sure about which substances they had consumed (20).

Thus, studies suggest that consumers of long-known substances of abuse might also be prone to NPS use. In the present multicenter study, we examined lifetime and recent NPS use in patients admitted to in-patient drug detoxification treatment for illicit drugs. NPS use was therefore investigated in the context of “classical” drug use, including the main substances used by members of the drug scene, as well as other long known substances such as LSD, Khat or psilocybin, etc.

## Methods

### Study Design

The present study used a prospective, cross-sectional design. Included were patients admitted to in-patient drug detoxification treatment in the German state of Nordrhein-Westfalen. Participating institutions were members of an association of facilities and professionals in the field of drug treatment and drug detoxification (Fachverband Qualifizierte stationäre Entzugsbehandlung Opiatabhängiger). In-patients undergo an anamnestic interview at admission to treatment, including questions concerning past and present drug use. For study purposes, the interview was standardized with regard to questions and answering formats, and specific questions about NPS use were included. Also basic socio-demographic characteristics were recorded (age, gender, migrant background, current relationship status, living with children, current employment). The laboratory analysis of the drug screenings routinely taken at treatment admission was expanded to include NPS. This required an additional transfer of urine samples to an external laboratory and was not carried out by all participating facilities (see below). The study was reviewed and agreed upon by the ethics board of the Medical Faculty, University Duisburg Essen. Data were collected during years 2018 and 2019.

### Sample Recruitment, Inclusion and Exclusion Criteria

All eligible patients admitted to drug detoxification treatment during a specified period (2 or 3 months, depending on the respective facility) were invited to participate. If patients agreed into participation, they were informed about the study aims and procedures and about data protection measures. In particular, patients were informed that their data were stored and analyzed in pseudonymous form only. Patients then gave their written informed consent.

Patients could be included into the study if they received a diagnosis of dependence from cannabis, opiates, cocaine or amphetamines. Exclusion criteria were: a patient did not understand German well enough to fully comprehend the study information and/or the interview questions; a patient showed cognitive impairments, including severe symptoms of intoxication or withdrawal, which prevented understanding of study information and/or the interview questions; a patient showed symptoms of a psychiatric disorder (e.g., acute psychosis) which cast doubt upon that s/he could fully understand the study information or could act freely; or a patient did not give informed consent.

Patients were informed that they could object to study participation at any time and without negative consequences, and that they could withdraw a given consent.

### Assessments

Patients were interviewed for consumption of psychotropic substances using a standardized questionnaire. It included names of substances or of types of substances, for example heroin, substances used in opioid agonist maintenance treatment (methadone, buprenorphine), cocaine, cannabis, alcohol, but also of substances with an assumed lower prevalence, such as opioid analgesics, gabapentin/pregabalin, muscarin, khat, ketamine, etc. For each substance or substance class, patients should indicate if they had ever used it at least once during lifetime. If yes, they were then asked about its consumption during the previous 30 days: on how many days, in which daily dose, and by which application route (intravenous, oral, nasal, by smoking/inhaling). Regarding lifetime consumption, patients were asked for lifetime frequency (< 5 times, 5 to 50 times, more than 50 times), historical years of regular consumption (defined here as at least weekly, e.g., every weekend), and historical years of daily or almost daily consumption.

Within the list of psychotropic substances, NPS were introduced as substances also known as “Legal Highs,” “Herbal Highs,” or “Research Chemicals.” In particular, four classes were listed: synthetic cannabinoids (“spice” etc.), i.e., substances which act like cannabis; synthetical stimulants (bath salts, mephedrone, etc.); Herbal Drugs (herbal ecstasy, etc.); and any other, not previously mentioned NPS. Patients were asked to give the name of consumed substances. In addition, they were asked whether there were particular reasons or circumstances when they consumed the respective NPS, for example because it could not be detected by urine tests, or served as a substitute for unavailable substances.

Comprehensiveness and practicability of the interview were tested in a pilot study with 12 patients, and questions and answering options were improved where necessary. The interviews were carried out by a resident from the respective ward.

### Analytical Testing

In addition to the interview, urine specimens routinely sampled at admission were sent to an external laboratory (LVR Klinik Viersen, head: Jürgen Sawazki). The determination of NPS as well as of common drugs out of a urine matrix was performed utilizing Solide-Phase-Extraction (SPE) and followed by a screening on a Gas Chromatography system coupled to a Time-of-Flight Mass spectrometer (GC-ToF-MS) (21). Acetate buffer and beta-Glucuronidase/Aryl Sulfatase were added to 3 ml urine and incubated for 30 min at 56°C. Afterwards the extraction of the basic drugs and Drugs-of-Abuse (DOA) was performed according to a validated method on a SPE cartridge. This step was followed by the injection of 1 µl of the extract into the GC-ToF-MS.

To perform a sensitive analysis the data were collected at a high detector voltage. A ToF system allows detecting every eluting analyte on a very high data rate. This allows the sensitive detection of very small amounts of psycho active substances. This is mandatory, since the NPS are excreted out of the body in small concentrations, the metabolites are excreted in an even smaller concentration however metabolites provide an important information on when the drug was abused and which drug was consumed. The separation of the mixture is performed in 14 min, followed with an automated deconvolution, which allows determining even coeluting substances.

In a library search the spectra of the drugs in the current analysis were then compared to the spectra of already entered drugs. Such libraries are commercial and for free available, but own spectra of relevant substances can be added. Such libraries contain hundreds of spectra of NPS and their metabolites (SWGDRUG.ORG). Possible structural modification of analytes can be determined, if the probability (match with the spectra in the library) is reduced. Additional research could be done for an unknown substance with the suspicion for NPS.

### Data Analyses

Completed interview forms and printouts of the urine analyses were pseudonymized using a code based on letters from a patient’s given name and his birthday. The documents were sent to the LVR Klinik Essen for data entry and statistical analyses.

## Results

### Self-Reports of NPS and Other Drug Use

Recruitment data refer to only 7 out of 8 participating sites; in one hospital, documentation of recruitment failed. During recruitment, 475 patients in the 7 hospitals were admitted to treatment with a dependence diagnosis for one or more illicit substances; 10.1% of these patients were excluded because of language problems, 10.7% because of or cognitive, psychiatric or substance related problems, and 22.1% refused participation or failed to give written consent. In addition, 2.7% did not complete the interview. Therefore based on 7 out of 8 participating sites, it is estimated that 54.3% of patients were finally included in the analysis.

In total, the eight study sites investigated n = 295 patients which could finally be analyzed. More than 4 out of 5 patients had an opiate related diagnosis (**Table 1**), and about half were transitions from opioid maintenance treatment, either for detoxification from concomitant substance use, or for detoxification from the maintenance drug. There were also high rates for diagnoses related to other substances of abuse. Rates of employment, living with partner, or living with children were low in this generally male sample.

**Table 1:** Sociodemographic characteristics and substance use diagnoses of the study sample (n = 295).

	
Female	21.2%
Male	88.8%
Age	Range, 19–64 years Mean, 39.0 years; SD, 8.4 years
Migrant background^1^	42.1%
Living with partner	16.9%
Living with children	13.1%
Employed full-time or part time	13.4%
Daily cigarette smoker	95.9%
**Substance use diagnoses (abuse or dependence)**	
Opiates	81.8%
Alcohol	38.7%
Cannabis	38.4%
Cocaine	34.0%
Benzodiazepines	25.9%
Amphetamines	12.1%

1 Patient and/or at least one parent was foreign born.

**Figure 1:**
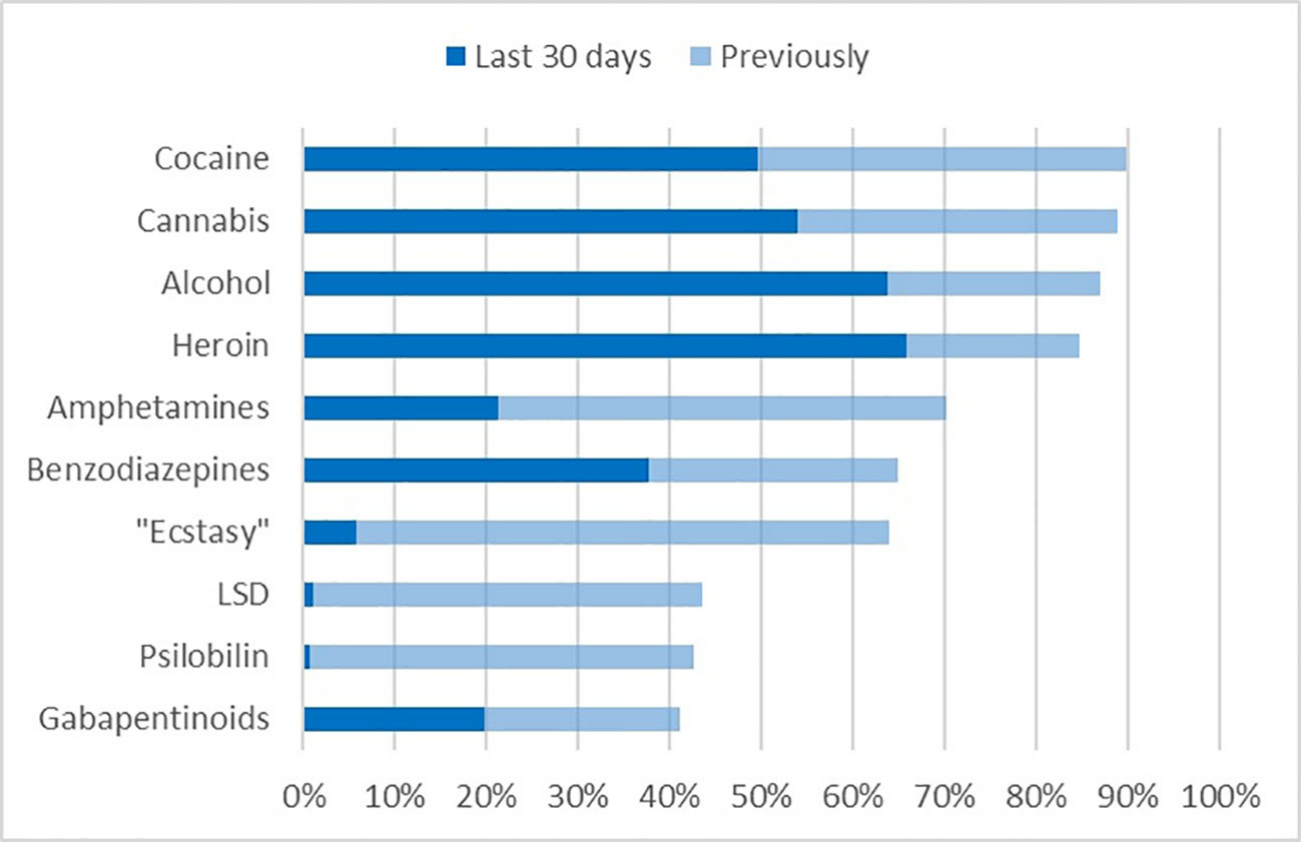
Top 10 of lifetime prevalence of substances of abuse, and their recent use.

In accordance with the substance related diagnoses, the vast majority of patients reported of lifetime use of heroin, but also alcohol, cannabis, cocaine, and amphetamines (**Figure 1**). These substances had also been used during 30 days before admission to detoxification treatment. Not counting nicotine, prescribed medications and maintenance drugs, more than one psychotropic substance during the last 30 days had been used by 86.0% of patients. The mean number of recently used substances was 3.3 (SD 1.8). Some substances with high lifetime prevalence (MDA/MDMA “Ecstasy,” LSD, and Psilocybin) had low or nearly zero prevalence during the last 30 days. Gabapentinoids, namely pregabalin, also belonged to the 10 substances with highest lifetime prevalence, and also had been used recently (20.0%) by a substantial proportion of patients. Note that the prescription of pregabalin for substance abusers is considered a malpractice in Germany.

**Figure 2:**
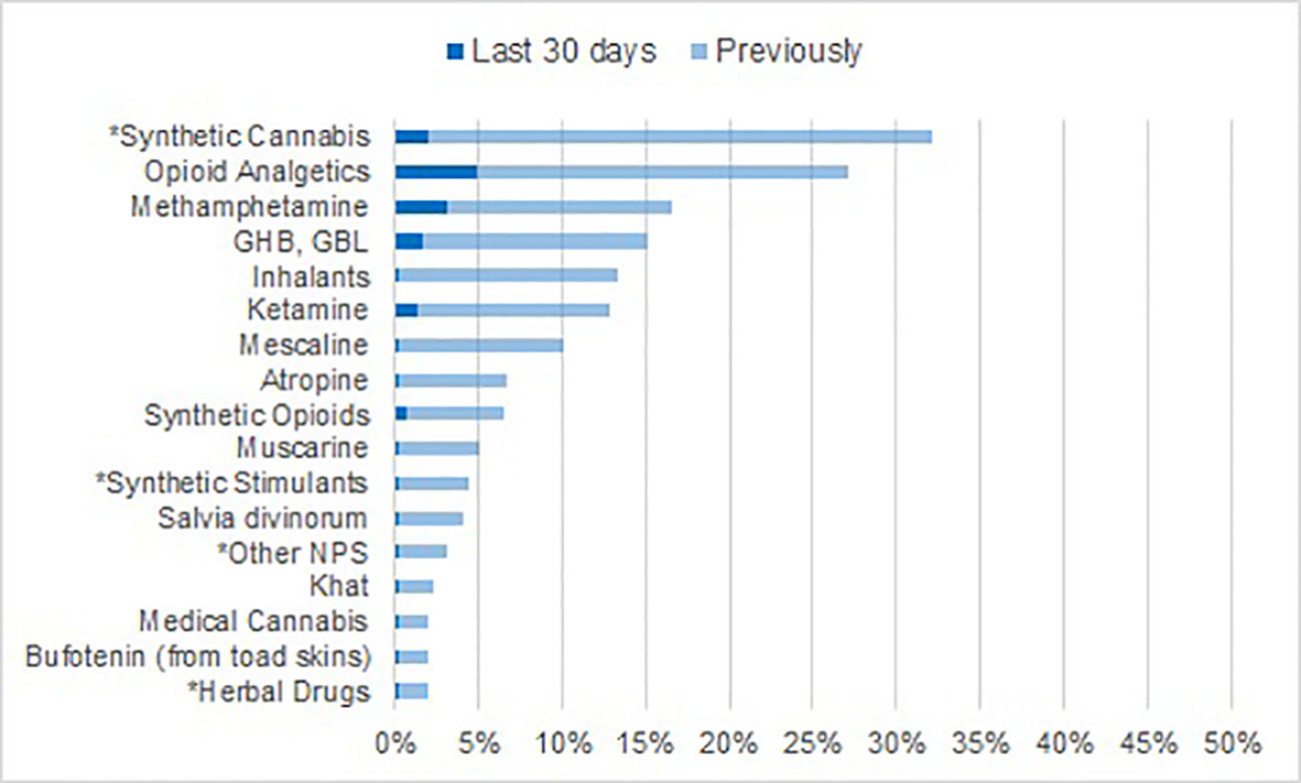
Lifetime prevalence and recent use of other substances including *NPS.

The proportion of patients with any self-reported lifetime NPS use was 32.6%. Almost all NPS users had consumed synthetic cannabinoids such as “spice” (32.1% of the total sample, **Figure 2**), synthetic stimulants had been used by 4.4%, other NPS (reported as “synthetic angel dust,” “synthetic ketamine,” 2CB, “micros,” “Armageddon,” or DMT) by 3.1%, and herbal drugs by 2.0%. The rate for synthetic cannabinoid use during the last 30 days was 2.0%, and 0.3% (1 patient) for each of the other NPS categories.

The total number of reports about consumed NPS was 126. Sixty-seven of these (53.2%) consisted of less than 5 episodes, 29 (23.1%) of 5- to 50, and 20 (15.9%) of more than 50 episodes. In 10 cases (7.9%) the number of episodes was not clear. There were 42 different patients (14.3% of the total sample) who had consumed NPS 5 times or more or for an unclear number of times during lifetime.

**Figure 3:**
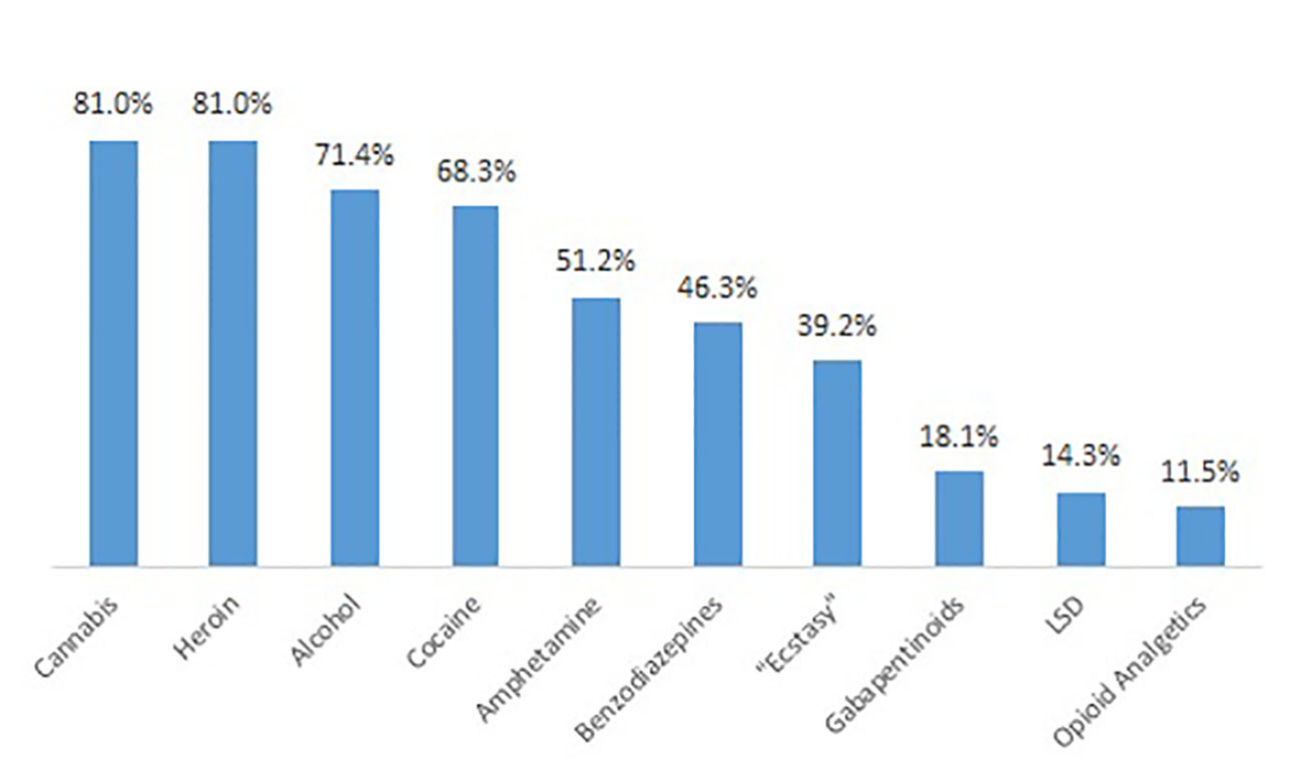
Top 10 of substances used regularly during lifetime.

**Figure 4:**
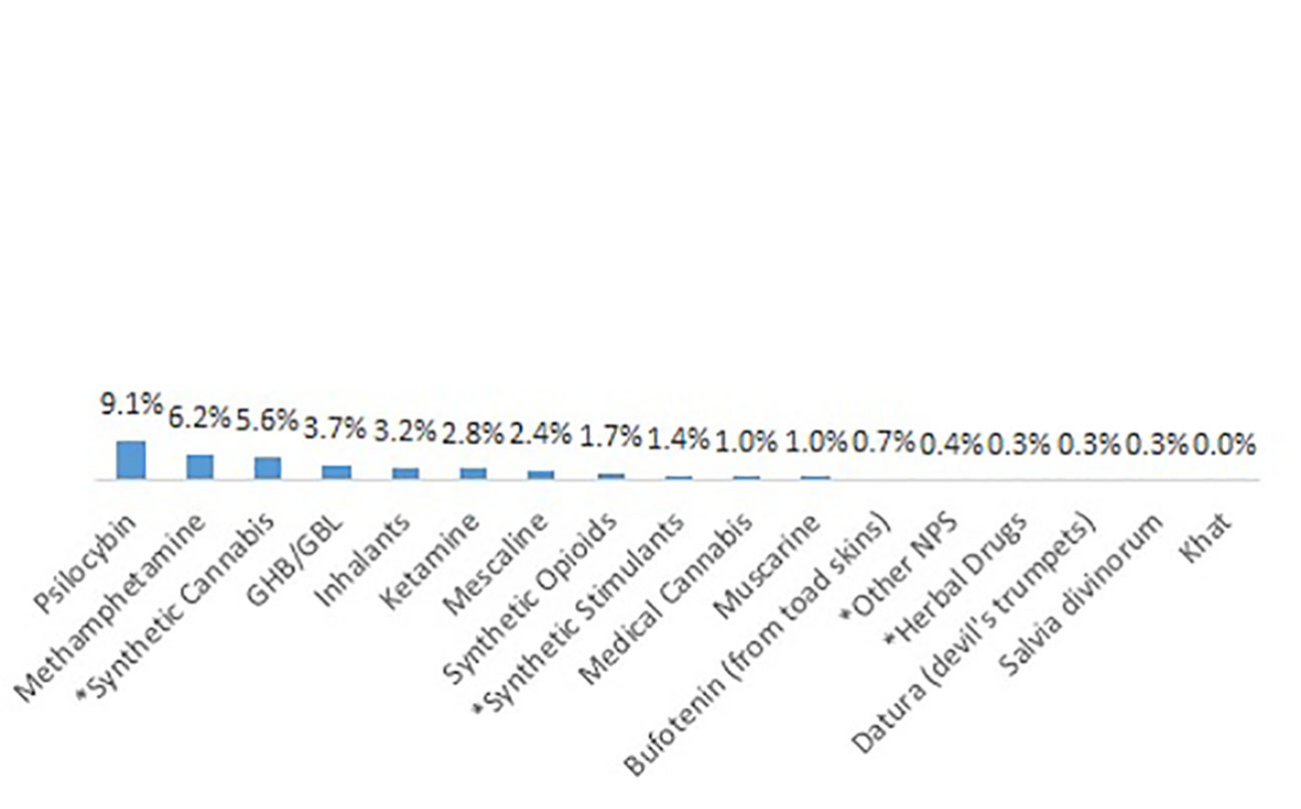
Other substances used regularly during ifetime, including *NPS.

Patients reported whether they had ever used a psychotropic substance regularly, i.e., at least weekly. Cannabis, heroin cocaine, alcohol, and heroin were the substances with the highest lifetime prevalence of regular consumption (**Figure 3**). Only a small proportion of patients reported of regular NPS consumption during lifetime (**Figure 4**); the most important type in this respect were synthetic cannabinoids (5.6%).

There were 77 statements about specific reasons or circumstances of NPS use. In only one case a pleasant psychotropic effect of the substance was mentioned as reason for consumption. Curiosity, exploration or an accidental offering were mentioned in 29 cases (37.6%). In 27 cases (35.1%), NPS were used in supervised surroundings, i.e prison (19 times, including 10 statements that the substance could not be detected by drug screenings there) or therapeutic settings (8 times). According to 5 statements (6.5%), NPS served as substitute for other drugs, and 4 statements (5.2%) were about non-detectability in drug screening, without further specification of the circumstances.

### Urine Analyses

**Figure 5:**
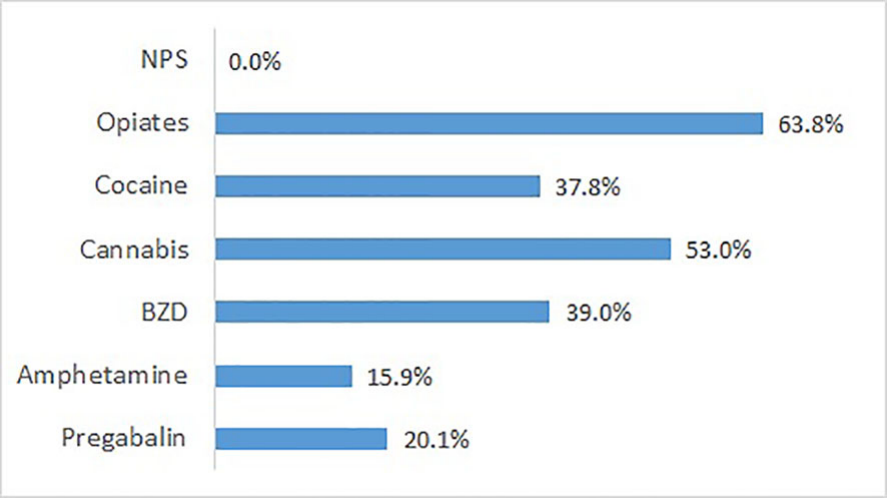
Substances found by GC/MS analysis of urines sampled at admission, except prescribed medication (n = 181 patients from five detoxification wards).

Additional urine analyses using a broadband GC/MS approach were carried out for patients from 5 of the participating sites (n = 181). Patient characteristics were very similar to the total group (mean age 39.1 years, 20.9% females, 39.2% migrant background, 82.3% with a diagnosis related to opioid use, 52.5% from opioid maintenance treatment). The pattern of results closely resembles that of self-reported substance use during the last 30 days (**Figure 5**). The substance class most frequently found was opiates (excluding opioids, i.e., maintenance drugs), followed by cannabis. NPS were found in none of the 181 samples. In addition, pregabalin was found in 20.1% of the samples. If an NPS had been used in the past, this was in more.

## Discussion

To our knowledge, the present study is the first to investigate the NPS use history of “classical” drug users in detoxification treatment. The present sample consisted of mainly male patients, mostly older than 30 years, and with a low employment rate. The vast majority was opiate dependent, and especially the rate of recent heroin use was high.

About one third of the sample reported of NPS lifetime use, but recent use was extremely rare. Moreover, more than half of NPS lifetime consumption consisted of less than 5 episodes, and about three fourth consisted of 1 to 50 episodes. Accordingly, rates for lifetime regular use (at least weekly) were very low. In accordance with self-reports, the GC/MS urine analyses did not produce any result positive for NPS.

The vast majority of used NPS were cannabimimetics, while synthetic stimulants and hallucinogens were infrequent. That does not mean that patients generally did not try out substances with such effects, as documented by the high rates of lifetime use of longer known substances, for example LSD, psilocybin, or MDMA (“ecstasy”). The markedly different lifetime use of these substances compared with NPS might in part be explained by the birth cohort of the present sample. When NPS appeared on the market, patients were mostly past their early 20s and perhaps beyond the age of experimenting with different substance classes. Nevertheless, according to patient reports, one third of NPS had been consumed out of curiosity, or to try out an unknown substance. In addition, nearly half of reported NPS were used in an institution (prison, or treatment facility) where drug use was sanctioned and NPS were not included into drug testing, and/or because it temporarily served as a substitute for other drugs.

The prevalence rate for recent NPS use in the present study was lower than in previous studies with other drug using groups, i.e., injecting drug users from Hungary and Scotland (15, 16), drug using patients in France (17), patients with substance use disorders in Scotland (4), or Swedish patients in opioid maintenance treatment (20). Moreover, cathinones are more prevalent in other studies (17). Regional or national differences between drug markets, including legal regulations, might play a role here. For example, in Switzerland NPS were rarely found in presentations to emergency rooms due to acute toxicity of psychoactive substances (21, 22) and were therefore considered not to be an important health issue, in contrast to other countries (23). Generally, members of a traditional drug scene dominated by opiate use in combination with cocaine, alcohol, cannabis, and/or benzodiazepines do not seem to prefer substances with hallucigenic properties. With regard to cannabimimetics, it could be argued that there is little reason for their continued use outside of prisons or treatment facilities, if cannabis, although controlled by the law, is easily available and its synthetic NPS versions are legally sanctioned. Moreover, many users experience psychological or health problems (9, 24), for example panic attacks, nausea, or circulatory problems (19), a fact that could further reduce demand for these substances.

So NPS had not been added to the range of “classical” substances of abuse in the investigated group. Instead, interview and urine data indicated an important role of pregabalin. In the last ten years, pregabalin has been extensively prescribed off-label for psychiatric conditions such as bipolar disorder, alcohol/narcotic withdrawal states, and ADHD, and it has entered the black market (25, 26). The principal population at risk consists of patients with other current or past substance use disorders, mostly opioid and multi-drug users who consume pregabalin in high dosages to achieve euphoria and also to reduce withdrawal symptoms, or to potentiate the effects of methadone (27). Pregabalin use was detected for example in 10.7% (28) and 14% (27) of hair specimens from of opioid maintenance patients in Italy, and in 4 to 17.7% of urine samples from opioid maintenance patients in Switzerland (29), Ireland (30), Sweden (20), and Israel (31), while one other study found zero prevalence in hair samples (32). The present finding indicates a constant or even increasing problem concerning pregabalin in the population of illicit drug users.

### Limitations

For determination of NPS use through face-to-face interviews we included several types and examples of NPS, and included the category “other NPS.” Such a combination of checklist and generic questions were found to reduce underestimation of NPS use (33). Still, it is conceivable that the actual prevalence of lifetime NPS use is higher than here determined, for example because patients are not always precisely aware about which novel substances they consume (20). Nevertheless, we assume that patients are aware of whether those substances which they consume frequently or regularly are NPS or not, so that we consider the very low rates for regular use and for frequent use as valid.

Another concern is the selection and self-selection of patients for this study. Findings only refer to patients who were not prevented from participation by language, psychiatric, cognitive, or drug-related problems (in total, more than one fifth of the patients admitted to drug detoxification), and who did not refuse to participate (also more than one fifth). It is unknown to us whether these groups of patients might show a distinct pattern of NPS (non-)use.

## Conclusions

NPS use in the studied population was mainly restricted to trial use or to temporary use in restricted contexts such as prisons. If such a consumption is considered worrisome, inclusion of NPS into urine drug screenings would probably further decrease consumption rate. It is possible that future cohorts of classic drug users will show higher rates of NPS, since the majority of the current sample had been in their late twenties or older when NPS emerged on the market and they were already used to other types of substances. Nevertheless, the profile of NPS, apart from cannabimimetics, seems not meet the preferences of the here studied patient group, e.g., drugs with hallucigenic properties are rarely used.

The high rate of pregabalin abuse in the current sample resembles findings in other countries with similar patient samples. This reminds us that legally prescribed drugs need to be closely monitored for their abuse potential. Other examples for this phenomenon are nonmedical use of benzodiazepines or of opiate analgesics.

## Data Availability Statement

The raw data supporting the conclusions of this article will be made available by the authors, without undue reservation.

## Ethics Statement

The studies involving human participants were reviewed and approved by Ethik-Kommission, Medizinische Fakultät der Universität Duisburg-Essen, Robert-Koch-Straße 9-11, D-45147 Essen, ethikkommission@uk-essen.de (file number 18-8580-BO). The patients/participants provided their written informed consent to participate in this study.

## Author Contributions

MS and NS designed the study, carried out the data analysis and interpreted the results. MS wrote the manuscript. JS and LS designed and carried out analyses of biological samples and wrote a description of the technical details. UB, MC-R, RS, HE, TK, BZ, and AN discussed the study design, organized the acquisition of data and contributed to the interpretation of results. The authors agree to be accountable for all aspects of the work in ensuring that questions related to the accuracy or integrity of any part of the work are appropriately investigated and resolved.

## Funding

The study was conducted as part of project JUSTSO, funded by the European Union’s Justice Programme — Drugs Policy Initiatives (number 806996 — JUSTSO — JUST-2017-AG-DRUG). The urine analyses were financially supported by Fachverband Qualifizierte stationäre Akutbehandlung Drogenabhängiger e.V.

## Conflict of Interest

The authors declare that the research was conducted in the absence of any commercial or financial relationships that could be construed as a potential conflict of interest.
