# Corrigendum: Prevalence of Novel Psychoactive Substance (NPS) Use in Patients Admitted to Drug Detoxification Treatment

**DOI:** 10.3389/fpsyt.2021.681542

**Published:** 2021-04-21

**Authors:** Michael Specka, Thomas Kuhlmann, Jürgen Sawazki, Udo Bonnet, Renate Steinert, Monika Cybulska-Rycicki, Helmut Eich, Benita Zeiske, Antje Niedersteberg, Luzia Schaaf, Norbert Scherbaum

**Affiliations:** ^1^LVR Hospital Essen Department of Addictive Behaviour and Addiction Medicine, Medical Faculty, University of Duisburg-Essen, Essen, Germany; ^2^Psychosomatische Klinik Bergisch Gladbach, Bergisch Gladbach, Germany; ^3^LVR Clinic Viersen, Viersen, Germany; ^4^Castrop-Rauxel Evangelical Hospital, Castrop-Rauxel, Germany; ^5^LWL-Klinik Münster, Münster, North Rhine-Westphalia, Germany; ^6^LVR Clinic Langenfeld, Langenfeld, Germany; ^7^Alexianer Hospital, Krefeld, Germany; ^8^Alexius/Josef Hospital, Neuss, Germany; ^9^LVR Clinic Düren, Düren, Germany

**Keywords:** novel psychoactive substances, pregabalin abuse, multiple substance use, drug dependence, drug detoxification, opiate dependents

In the published article, there was an error in affiliation **7** Instead of “**Clinic Maria Hilf GmbH, Moenchengladbach, Germany**” it should be “**Alexianer Hospital, Krefeld, Germany**.”

The authors apologize for this error and state that this does not change the scientific conclusions of the article in any way. The original article has been updated.

